# Challenges and Perspectives on Breast Cancer Survivorship: The Journey Continues

**DOI:** 10.1093/geroni/igaa057.317

**Published:** 2020-12-16

**Authors:** Victoria Raveis, Simona Kwon

**Affiliations:** 1 New York University, Maplewood, New Jersey, United States; 2 NYU School of Medicine, New York, New York, United States

## Abstract

Women have a 1-in-8 lifetime risk of breast cancer. Earlier diagnosis and treatment advances have improved 15- and 20-year survival rates. Increased survival can mean coping with the effects of cancer and its treatment over an extended period of time, while experiencing age-related changes in functioning and the emergence of other health issues. To explore breast cancer survivors’ perspectives on their issues and concerns across the life-course, focus groups were conducted with a culturally diverse sample (N=18) of survivors (72% white, 28% Black, 11% Hispanic). Participants were 44-82 years old. Most, 83% were 50 and older, 56% were 60 and older. The majority (83%) were diagnosed in their 40’s and 50’s. Two were diagnosed in their early 30’s and one at age 68. Participants reaffirmed the necessity, as a breast cancer survivor, of being a life-long health advocate on their own behalf, and the importance of being self-informed. As one woman commented: “Knowledge is power”. Survivors shared that their emergent health issues were complicated by their cancer history, and, that, as a cancer survivor, “I never stop worrying”. A widespread concern was not knowing if the health issues and co-morbidities they experienced (such as joint pain, neuropathy, tendinitis, heart disease), were age-related, a consequence of their cancer, or a late treatment effect. An overriding sentiment expressed was that clinicians have not recognized the importance of quality of life in cancer survival. As a survivor succinctly stated: “We are living longer, but we need to live long with quality of life.”

